# Book Reviews

**Published:** 1873-11-15

**Authors:** 


### Book Reviews.

Chemistry, Inorganic and Organic, with Experiments. By Charles Loudon Bloxam. With 295 illustrations. From the Second and Revised English Editions. Philadelphia: Henry C. Lea. (For sale by Jansen, McClurg & Co., Chicago.)

When the first edition of this work appeared, a few years since, its sterling merits won for it a wide recognition and a justly enviable reputation; this second edition, which has just appeared—revised, enlarged, and brought down to date—cannot fail to add materially to the high esteem in which the book is already held.

Of all the numerous works upon elementary chemistry that have been published within the last few years, we can point to none that, in fulness, accuracy and simplicity, can surpass this; while in the number and detailed description of experiments, as also in the profuseness of its illustrations, we believe it stands above any similar work published in this country. To the teacher of chemistry, these accurate descriptions of such a large number of experiments—many of them quite unique—will be found of especial value; while to the student of the science they afford a highly profitable source of instruction and an inestimably valuable means for the explanation of obscure points. We cannot speak too highly of many portions of the work, and especially of the chapter devoted to carbon and its compounds. The statements made are clear and concise, and every step proved by an abundance of experiments which excite our admiration as much by their simplicity as by their direct conclusiveness.

We have observed, with great disfavor, the growing tendency manifested by recent chemical writers to transfer chemistry, in part at least, from its natural position among the experimental sciences to a very doubtful place among the mathematics. Some of our most popular chemical works of the present day devote a very considerable space to purely theoretical and algebraic considerations, abridging, of course, by just so much, the far more valuable, practical and experimental parts of the science. This tendency, we think, cannot be too greatly deprecated; for we believe the distaste for chemistry which of late years has shown itself so painfully among most of our college students, may be traced, to a great extent, to this irrational attempt to change the natural domain of the study. We therefore give a doubly hearty welcome to a work which, like Prof. Bloxam's, places chemistry in its true and valuable position as a practical and experimental science—which avoids the lengthy discussions of purely theoretical questions, and thus commends itself at once to the numerous members of the medical profession, and the public in general, who desire for a text-book, or book of reference, a work not occupied with theory, but filled with important facts.

We are pleased to find that the author continues to adhere to the use of the ordinary English weights and measures, and the Fahrenheit thermometer. However perfect the decimal system may be, it is so little understood by the majority of readers at the present time that its use renders almost unintelligible any statements involving its employment. The busy practitioner, especially, who rarely has the time to convert *grammes* into grains, or *litres* into pints, by mathematical calculations, will duly appreciate the value of a work in which no such calculations are necessary.

We praise the work under consideration highly, for we believe that its real merits demand such a recognition; yet, in one or two minor points, we think it is open to a little adverse criticism. There are a number of almost inexcusable omissions, respecting various substances, which we are at a loss to explain. Although ozone is spoken of at considerable length, its companion, ant-ozone, is not even mentioned, notwithstanding its existence has been positively proved, and it is known to be possessed of some remarkably interesting properties. Two pages, at least, could be profitably devoted to the description of this remarkable allotropic form of oxygen, and yet its name is not even mentioned. So, too, nothing is said concerning the oxy-chloride of zinc, although its important applications in the arts, and especially in dentistry, certainly demand for it a brief description at least.

A few other less important omissions might be mentioned; yet candor compels us to acknowledge that, in the main, the book is remarkably full.

We are surprised to find that Prof. Bloxam, in this new and revised edition, still continues to use the old system of nomenclature, and, to a great extent, the old system of notation: calling epsom salts, sulphate of magnes*ia;* chalk, the carbonate of *lime;* writing sulphuric acic, $SO_{31}$; and so on. The author evidently believes that facts justify his adherence to the old system; but the mere fact that the vast majority of chemical authorities have long since deserted it, strongly indicates that there are advantages in the new system of nomenclature and notation that cannot be gained by the old; and the author himself tacitly acknowledges this, in part, by using occasionally the new formulæ.

Laying aside, however, these few exceptions that we take to the work, we can unhesitatingly praise the book, and confidently commend it to the favor of the public as possessing, on the whole, in our opinion, more true merits than any other chemical work of a similar size that can be found in the market. W. S. H.

A Practical Treatise on the Diseases of the Ear, including the Anatomy of the Organ. By D. B. St. John Roasa, M.A., M.D. New York: Wm. Wood & Co. (For sale by Jansen, McClurg & Co., Chicago.)

This is a volume of 530 pages, intended as a guide for those who wish to treat the diseases of the Ear. The topics are considered under the following headings: Anatomy of the Auricle and External Auditory Canal.—The Examination of Aural Patients.—Diseases of the Auricle.—Diffuse and Circumscribed Inflammation of the External Auditory Canal.—Parasitic Inflammation of the External Auditory Canal.—Inspissated Cerumen.—Foreign Bodies in the Ear.—Anatomy of the Middle Ear.—Injuries of the Membrana Tympani.—Acute Catarrhal Inflammation of the Middle Ear.—Chronic Non-Suppurative Inflammation of the Middle Ear.—Acute Suppuration of the Middle Ear.—Anatomy of the Internal Ear.—Diseases of the Internal Ear.—Deaf Muteism and Hearing Trumpets.

Considerable space is given to illustrative cases, with a view of showing the actual symptoms of Aural diseases, and the results of treatment.

The subjects seem to be treated of in a complete as well as practical manner, and the text is illustrated by numerous wood-cuts.

Chromo-lithographic illustrations of eight different diseased conditions, as revealed by the otoscope, are also given in a plate at the end of the volume.

A Manual of Medical Jurisprudence. By Alfred S. Taylor, M.D. Seventh American edition, revised from the author's latest notes, and edited by John J. Reese, M.D. Philadelphia: Henry C. Lea. (For sale by Jansen, McClurg & Co., Chicago.)

This well-known standard work on Medical Jurisprudence is here presented to us in a new and revised edition. From the author's preface it appears that the principle changes made in the present edition consist in the addition of two chapters on Evidence and Duties and Responsibilities of Medical Witnesses, and the introduction of numerous engravings representing the crystalline forms of poisons, etc., and the apparatus used for their detection. Some medico-legal subjects, not hitherto treated in the previous editions, have been also introduced. The additions made by the editor amount to about one hundred pages.

An Introduction to Practical Chemistry, including Analysis. By John E. Bowman, F.C.S. Edited by Charles L. Bloxam, F.C.S. Philadelphia: H. C. Lea. (For sale by Jansen, McClurg & Co., Chicago.)

This is the sixth American edition of this little work, reprinted from the new revised English edition.

The Student's Guide to Medical Diagnosis. By Samuel Fenwick, M.D., F.R.C.S. From the third revised and enlarged English edition, with eighty-four illustrations on wood. Philadelphia: Henry C. Lea. (For sale by Jansen, McClurg & Co., Chicago.)

This is a small volume, of 328 pages. It covers the ground of Medical Diagnosis in a concise, practical manner, well calculated to assist the student in forming a correct, thorough, and systematic method of examination and diagnosis of disease. The illustrations are numerous and finely executed. Those illustrative of the microscopic appearance of morbid tissues, etc., are especially clear and distinct.

The Physician's Visiting List for 1874. Twenty-third year of its publication. Philadelphia: Lindsay & Blakiston. (For sale by Jansen, McClurg & Co., Chicago.)

Transactions of the American Medical Association. Vol. 23. 1872. (For sale by W. B. Keen, Cooke & Co.)

